# The Survival and Treatment of *Fusarium oxysporum* f. sp. *cubense* in Water

**DOI:** 10.3390/jof7100796

**Published:** 2021-09-24

**Authors:** Sahabne Ullah, Diane Mostert, Kobus Serfontein, Altus Viljoen

**Affiliations:** 1 Department of Plant Pathology, Stellenbosch University, Stellenbosch 7602, Western Cape, South Africa; shoshiullah@gmail.com (S.U.); altus@sun.ac.za (A.V.); 2 ICA International Chemicals, Stellenbosch 7601, Western Cape, South Africa; kobus@icaonline.co.za

**Keywords:** Fusarium wilt, water, survival, treatment, chlorine, ozone, UV peracetic acid

## Abstract

*Fusarium oxysporum* f. sp *cubense* (Foc), the causal agent of Fusarium wilt, is one of the most devastating constraints to banana production worldwide. The spread of Foc in water is particularly concerning, as infested water can rapidly contaminate disease-free areas. The objectives of this study were to investigate the survival of Foc in water and to test the effectiveness of water treatment with chlorine, ozone, UV, and peracetic acid. The study indicated that Foc spores can survive in water for more than 120 days, but that viability was reduced in stagnant water, probably due to anaerobic conditions when spores settled at the bottom. It is therefore recommended that surface water be extracted and treated before it is used for irrigation. The efficacy of all water treatments was reduced in the presence of soil, implying that water needs to be soil-free before treatment. The use of peracetic acid is recommended to treat Foc-contaminated water, as it is safe for use and does not require installation costs although it is effective at treating Foc-contaminated water, ozone would require significant input costs and chlorine can produce harmful disinfection by-products. UV would be impractical for field application because of the high doses required to eliminate Foc.

## 1. Introduction

Fusarium wilt of banana is caused by the soil-borne fungus *Fusarium oxysporum* f. sp. *cubense* (Foc), a plant pathogen considered to be one of the most destructive in agricultural history [1]. When introduced into a plantation, Foc can cause massive losses and is almost impossible to eradicate, as the fungus can survive in soil for decades [2,3]. The pathogen is dispersed with infected planting material and with infested soil attached to shoes and farming equipment. It is also rapidly disseminated with contaminated water used for irrigation, and in run-off water from infested fields after flooding [4]. Spread of Foc can be prevented by using clean planting materials and irrigation water, and the proper sanitation of shoes, vehicles and field equipment. The only method to manage Fusarium wilt in infested fields is to replace susceptible with resistant banana varieties [5].

Water is an essential resource for productive banana farming. A banana plant obtains water from natural precipitation in the wet tropics and from sprayer or flood irrigation in dryer areas. Tropical storms, cyclones and typhoons are common events that swamp banana fields, causing damage to plants and infrastructure, which can also contribute to the spread of diseases. When contaminated water is used for irrigation or water flows from infested to Foc-free areas, the pathogen may contaminate banana production extensively [6]. For instance, Fusarium wilt was reported to rapidly increase after heavy rainfall in both the Philippines [7] and Mozambique [8]. The irrigation of banana plantings with contaminated water from the Pearl River in China [9] was also reported to have significantly contributed to the spread of Foc tropical race 4 (TR4) [10].

Information on the survival and spread of Foc in water is limited. Stover [1] indicated that the survival of Foc was reduced in infested soils approximately 40–50 days after flooding [11], and that inoculum levels in the top 2–5 ft layer of soil was decreased for a period of 18 months. However, two years after treatment losses increased again to levels that prevented banana production [1,12]. Laboratory tests done by Stover [13] showed that Foc survived for up to 165 days in soil submerged under 2.5 cm of water. It was further reported that flowing water allowed aeration, thereby enabling the pathogen to survive 70 days longer than in stagnant water [13]. Chlamydospores were produced when soil was flooded with water and was exposed to an atmosphere of carbon dioxide [14]. These studies concluded that oxygen was needed for Foc to survive in flooded soils.

To reduce the spread of *Fusarium* spp. in water, treatments could involve filtration [15,16], non-chemical treatments with heat [17] and ultraviolet light (UV) [18], or chemical treatments with ozone [19,20], chlorine [21,22], peracetic acid (PAA) [23,24] and hydrogen peroxide [25,26]. The efficacy of water treatments is influenced by the chemical characteristics, temperature, pH and the presence of other microbes and particulate matter in the water [15]. The application rate and contact time of water therapies will not necessarily be effective against all pathogens and for all water qualities [22]. Therefore, the efficacy of different treatments of water contaminated with Foc, needs to be investigated.

Ozone is considered one of the most popular water treatments and has been used to treat wastewater, drinking water, and for the post-harvest treatment of pathogens on many crops [27,28]. It oxidizes the cell membrane, DNA, RNA and proteins of a target organism, thereby resulting in cell death. As ozone decomposes, it forms hydroperoxyl and hydroxyl radicals, which also act as oxidizing agents [29]. Ozone has shown its efficacy against conidia of *F. oxysporum* at a concentration of 0.6 ppm for 3 min [20]. However, when applied to dam water contaminated with *F. oxysporum*, a concentration of 1.1 ppm was required at 4 min for disinfection [19]. Ozone is a useful product as it requires low dosages and short exposure times for disinfection due to its high oxidation potential [30]. It is also environmentally friendly and leaves no harmful residues [29]. Its disadvantages include high installation and maintenance costs [31].

Chlorine is also commonly used to treat irrigation water. It can be applied either as chlorine dioxide gas or as hypochlorite salts [32]. Both these forms oxidize organic material, but chlorine dioxide is more potent than hypochlorite salts [18]. The efficacy of chlorine treatments is greatly influenced by the amount of organic material and the pH in water. More organic matter in water makes chlorine less effective. Hypochlorous acid, which is formed when chlorine is applied as hypochlorite salts, interacts with water and is most efficient at a pH between 6.5–7. As the pH of water increases, the hypochlorous acid is converted to hypochlorite, which is a weaker oxidizer and disinfectant. Chlorine gas is also affected by pH but is effective over a wide pH range (4–10). Chlorine is readily available and relatively cheap, easy to use and requires no installation or maintenance [33]. Sodium hypochlorite applied at high concentrations (0.6–50%) has been found to be effective against Foc as a sterilant [34,35,36]. The treatment of irrigation water at such rates, however, would have a negative impact on the environment due to disinfectant by-products (DBP). For instance, a chlorine concentration limit of 0.25 ppm is recommended for the disinfection of wastewater used for irrigation in South Africa [37]. Chlorine was effective for the treatment of *F. oxysporum* conidia in water at 5–10 ppm for 0.5–1.5 min. When applied to chlamydospores in water, an exposure time of 20 min was required, but in dam water this increased to 30 min [22].

Chemical disinfection with PAA products is facilitated through two active ingredients, acetic acid and hydrogen peroxide [38], which are commonly used in the pharmaceutical, beverage, paper, and food industries [39]. The disinfectant can oxidize enzymes and proteins that can affect the transport of ions between membranes [40] and can induce mutations in DNA [41]. The efficacy of PAA products against Foc has not been tested, and appropriate concentration and exposure time for the treatment of other *F. oxysporum* species has not been established. For *F. oxysporum* f. sp. *narcissi*, a concentration of 0.5% was effective at an exposure time of 80 min [23], while for *F. oxysporum* on watermelon a concentration of 80 mg/L was effective at an exposure time of less than 5 min [24]. PAA products produce a limited amount of DBP and is thus an environmentally friendly product [42]. It is also effective at pH values of 5–8, but its efficacy is negatively affected by organic material [43].

UV radiation is a very common non-chemical water treatment option. The energy discharged from UV light reacts with DNA and RNA of target organism and eliminates the ability of pathogens to be infectious [22]. The duration and intensity of water treatment will determine the efficacy of UV radiation, as UV energy is absorbed by particles in the water, water quality, turbidity and the amount of organic material present. UV treatment of *F. oxysporum* in water reported that a dosage of 70–250 mJ·cm^−2^ is required to eliminate the fungus [22,44]. An advantage of UV radiation is that it is environmentally friendly, while its drawbacks include high installation and maintenance costs. It is also ineffective in the presence of soil, with filtration before treatment resulting in additional costs [18].

The objective of this study was to determine the survival of Foc in still and agitated water, both in the presence and absence of soil. The efficacy of ozone, chlorine, a PAA product, and UV for the decontamination of water infected with Foc was also investigated.

## 2. Materials and Methods

### 2.1. Fungal Isolates

Isolates of Foc race 1 (CAV 2123 and CAV 2260), Foc subtropical race 4 (STR4) (CAV 95 and CAV 115) and Foc TR4 (CAV 2307 and CAV 3049) were used in this study. The isolates are all stored in the *Fusarium* culture collection at the Department of Plant Pathology at Stellenbosch University in South Africa.

### 2.2. Preparation of Foc Inoculum

Foc inoculum were produced in 250 mL Erlenmeyer flasks as follows. Each flask was filled with 62.5 g potting soil (Reliance, Paarl, South Africa) and 250 mL of distilled water, which was agitated overnight at 90 revolutions min^−1^ (rpm). The soil suspensions were then filtered through a 2 mm mesh sieve to remove large soil particles, and the filtrate strained through eight layers of cheesecloth. One hundred mL of each filtrate was then transferred to 250 mL Erlenmeyer flasks. The pH of the filtrate was adjusted to 7, and 2.5 mg glucose was added to each flask. The soil substrate was then autoclaved for 20 min at 121 °C on two consecutive days. After the soil substrate was cooled down, 4 mg of streptomycin sulphate (Merck KGaA, Darmstadt, Germany) was added to each flask and the flasks were left at room temperature. After 3 h, 50 mL of the soil suspension was poured into sterile 250 mL tissue culture flasks (Greiner Bio-One GmbH, Cellstar, Johannesburg, South Africa) and inoculated with either Foc race 1, STR4 or TR4 isolate. Three flasks were inoculated with each isolate, and three flasks with a sterile soil suspension were used as controls. The flasks were then incubated on a shaking incubator rotating at 90 rpm (Labcon, Petaluma, CA, USA) in the dark for one month at 25 °C.

### 2.3. Survival of Foc

To determine the survival of Foc in water, four buckets were filled with 20 L of distilled water and incubated in the dark at 25 °C. One kg of soil was added to two of the four buckets, and the water stirred to suspend and equally distribute the soil (Figure S1). An air pump (ViaAqua VA-130A, Cape Town, South Africa) was placed at the centre of two of the four buckets, one with soil and the other without soil, and switched on to continuously stir the water. Foc TR4, ST4 and race 1 were used to prepare a 10^3^ spores mL^−1^ spore mixture and then added to each of the four buckets. The survival of Foc in water was determined at 1, 7, 14, 30, 60 and 120 days after inoculation. Samples were collected by extracting 100 mL of water from the top, centre and bottom of each bucket with a fast pipette (Labnet International Inc., Edison, NJ, USA), and depositing these water samples into sterile 250 mL Erlenmeyer flasks. From each flask, 500 µL aliquots of the spore suspensions were then plated onto 10 Petri dishes with potato dextrose agar (PDA) modified with 0.04 gL^−1^ of streptomycin sulphate (PDA+). The PDA+ plates were incubated at room temperature, and the number of colony forming units (CFU) counted after two days. The whole experiment was repeated.

### 2.4. Water Treatments

#### 2.4.1. Foc Inoculum

The effect of different water treatments was tested on a spore suspension mixture of Foc TR4, STR4 and race. For all treatments the spore suspensions were diluted to 10^3^ spores mL^−1^ before treatment.

#### 2.4.2. Ozone

The effect of ozone was tested on Foc using an ozone generator (Del Ozone Genesis, San Louis Obispo, CA, USA). Forty mL samples containing Foc spore suspensions in the presence and absence of soil were prepared in 50 mL Falcon tubes. The tubes were then exposed to 3 g h^−1^ ozone for 10, 30 and 60 min. Positive controls were not treated with ozone. The tubes were left open for 1 h after treatment to allow the ozone to dissipate, and 500 µL of the suspensions were then pipetted onto five PDA+ plates. The plates were incubated for two days and the number of CFU’s counted, and the experiment was then repeated.

#### 2.4.3. Ultraviolet Light Radiation

Water with and without soil, containing a Foc spore suspension, was treated with UV at a dose of 100, 200 and 300 mJ.cm^−2^ by using a low-pressure UV lamp (Berson, Nuenen, The Netherlands). The Foc spore suspension (40 mL) was first placed in a 50 mL beaker and the water quality was measured using a UV transmission (UVT) meter (Berson, Nuenen, The Netherlands). Before treatment, the UV light was switched on for 10 min to measure the wavelength using a radiometer (International light technologies ILT1400, Peabody, MA, USA). Once the UVT and wavelength were measured, the exposure time was calculated as described by the equation below:

(1) $$I_{\mathrm{avg},\lambda \;(\mathrm{mW}{\cdot \mathrm{cm}}^{-2})}{=I}_{0}\lambda \;\left[\frac{{1-e}^{\mathrm{dln}(\mathrm{UVT}\left(\lambda \right))}}{-\mathrm{dln}(\mathrm{UVT}\left(\lambda \right))}\right]$$

(2) $$\mathrm{Desired}\;\mathrm{dose}\;\left(\mathrm{mJ}{\cdot \mathrm{cm}}^{-2}\right)=\mathrm{Average}\;\mathrm{intensity}\;\left(\mathrm{mW}{\cdot \mathrm{cm}}^{-2}\right)\;\times \;\mathrm{Exposure}\;\mathrm{time}\;(s)$$

In the above equation, I_(avg,λ)_ refers to the average intensity of UV light over the sample depth (d) in cm; UVT(λ) refers to the UV transmission of the sample at a wavelength (λ) of 254 nm, determined by using an optical path length of 1 cm; I_0_(λ) is the intensity of UV light measured at the surface of the sample. The exposure time was calculated by dividing the desired dose by the average intensity.

Afterwards, the exposure time was calculated for each dose. A stirrer bar was added into each beaker containing the Foc suspension and placed on a magnetic stirrer under UV light. Once the exposure time was reached, 500 µL from each beaker was pipetted onto five PDA+ dishes and incubated at room temperature and CFUs were counted after two days. The positive control sample was not treated with UV radiation. The experiment was repeated.

#### 2.4.4. Chlorine

The effect of chlorine on Foc was tested by adding 0.1 g of granular pool chlorine (HTH, Lonza, Johannesburg, South Africa) to 20 L of distilled water in the presence and absence of soil and the pH was adjusted to 7. After 10, 20 and 30 min, 100 mL of the water was collected from the top of the bucket using a fast pipette and immediately treated with 100 mL of an inactivator (0.5 g monopotassium phosphate, 0.5 g sodium citrate, 8.0 g sodium taurocholate, 1.5 g sodium thiosulfate, 8.0 g polyoxyethylene sorbitan monooleate in 1 L of distilled water) in a 250 mL Erlenmeyer flask. Aliquots of 500 µL from each sample were then transferred onto five PDA+ dishes, and the Petri dishes were incubated for two days, whereafter the CFU’s were counted. Non-treated water with Foc was used as the positive control, and the experiment was repeated.

#### 2.4.5. Peracetic Acid

HyperCide^®^ (ICA International Chemicals, Stellenbosch, South Africa) (14% PAA and 22% hydrogen peroxide) at a dose of 5 mL L^−1^ was used to treat Foc spore suspensions in the presence and absence of soil. After 10, 20, 30, 60 and 120 min, the treatments were inactivated by decanting the solution into 40 mL of an inactivator media (described earlier) in 250 mL Erlenmeyer flasks. Water that was not treated with the PAA product was used as the positive control. From each sample, 500 µL was transferred onto five PDA+ plates, the plates were incubated and the CFUs were counted. The experiment was repeated.

### 2.5. Statistical Analysis

SAS^®^ version 9.4 software (SAS Institute Inc., Cary, NC, USA) was used for Leven’s test for homogeneity, Shapiro–Wilk’s test for normality, and ANOVA to check for significant differences between treatments. A 95% least significant difference was used to make pairwise comparisons using Tukey’s test. For testing the survival of Foc in water, ANOVA was initially performed to determine whether the different factors (day, motion, the presence and absence of soil, depth) had a significant effect on each other. These factors were then analysed separately. For the water treatment experiments, ANOVAs were performed for each treatment separately. Colonies that were too many to count were given a maximum value of 300 CFUs.

## 3. Results

### 3.1. Survival of Foc in Water

**Figure 1:**
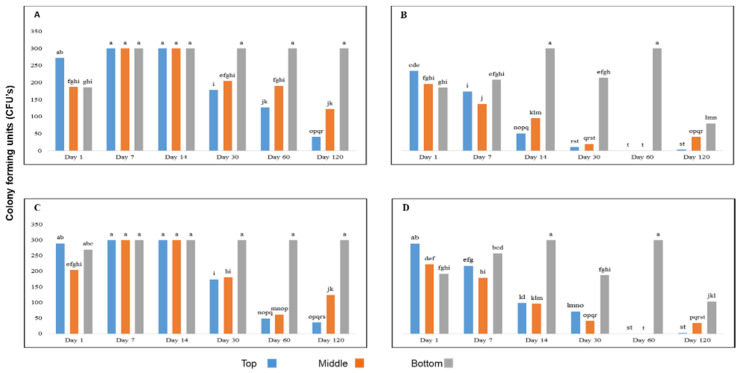
The effect of sampling depth, motion and the presence and absence of soil on the survival of Fusarium oxysporum f. sp. cubense in water. (**A**) Agitated water in the absence of soil, (**B**) Still water in the absence of soil, (**C**) Agitated water in the presence of soil and (**D**) Still water in the presence of soil. Significant differences (*p* ≤ 0.05) according to Tukey’s test are indicated by different letters.

Significantly more Foc spores survived in water that was agitated than in still water regardless of whether soil was present or not (Figure 1). There were also significantly more CFUs surviving near the surface and in the middle of the water in the first 14 days after inoculation than after 30 days (Figure 1), indicating fewer colony forming units survived in water over time. When water was agitated, more colony forming units survived on day 7 and day 14 in the presence and absence of soil at all depths compared to later dates (Figure 1A,C). The colonies at the bottom of the buckets did not decrease between days 7 and 120 when water was agitated but was reduced in still water after 120 days (Figure 1A,C). The number of CFUs collected near the surface and in the middle of still water was reduced after seven days, but those surviving at the bottom were high until day 60, whereafter it dropped at day 120 (Figure 1B,D).

### 3.2. Water Treatments

#### 3.2.1. Ozone

**Figure 2:**
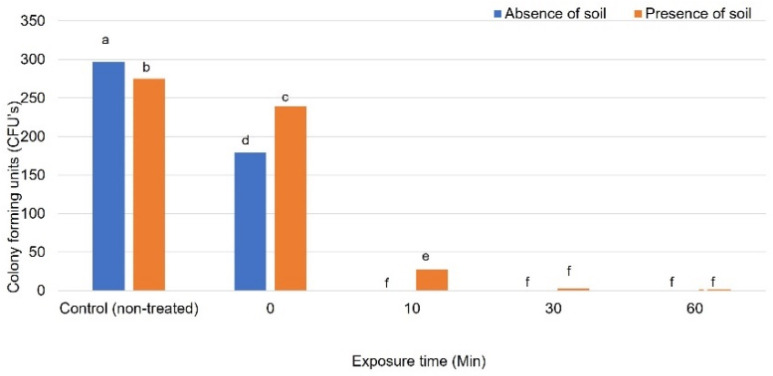
The survival of *Fusarium oxysporum* f. sp. *cubense* in water in the presence and absence of soil after treatment with ozone. Significant differences (*p* ≤ 0.05) according to Tukey’s test are indicated by different letters.

Ozone significantly reduced Foc CFUs in clean water after treatment and completely eradicated the pathogen after 10 min (Figure 2). In contrast, Foc CFUs were only eradicated after 30 min in water containing soil, even though it was significantly reduced at earlier time points compared to the untreated control (Figure 2).

#### 3.2.2. Chlorine

**Figure 3:**
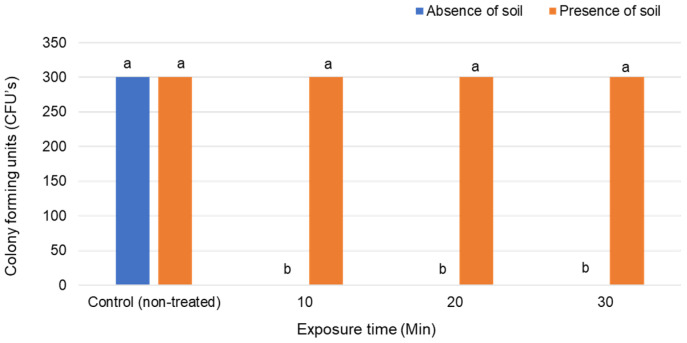
Treatment of *Fusarium oxysporum* f. sp. *cubense*-contaminated water in the presence and absence of soil with granular pool chlorine. Significant differences (*p* ≤ 0.05) according to Tukey’s test are indicated by different letters.

Chlorine eradicated Foc in clean water within 10 min but did not affect the fungus in water with soil (Figure 3). The survival of Foc in water with soil was similar to that in the control treatments (Figure 3).

#### 3.2.3. Peracetic Acid

**Figure 4:**
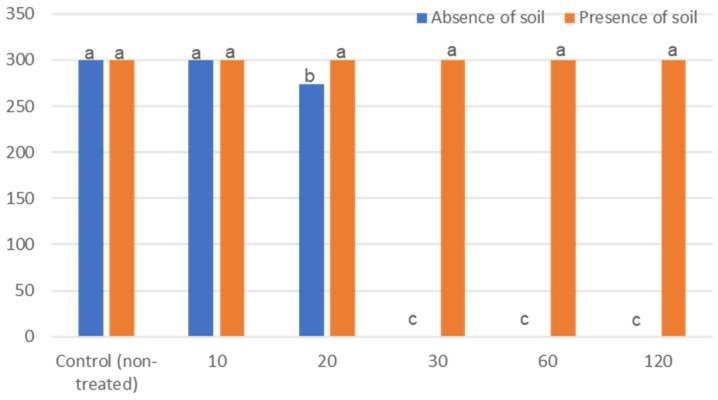
Treatment of *Fusarium oxysporum* f. sp. *cubense*-contaminated water in the presence and absence of soil with peracetic acid. Significant differences (*p* ≤ 0.05) according to Tukey’s test are indicated by different letters.

Foc product was significantly reduced after 10 min with PPA in the absence of soil, and was eradicated after 30 min. In the presence of soil, the PAA product was ineffective (Figure 4).

#### 3.2.4. Ultraviolet Light Radiation

**Figure 5:**
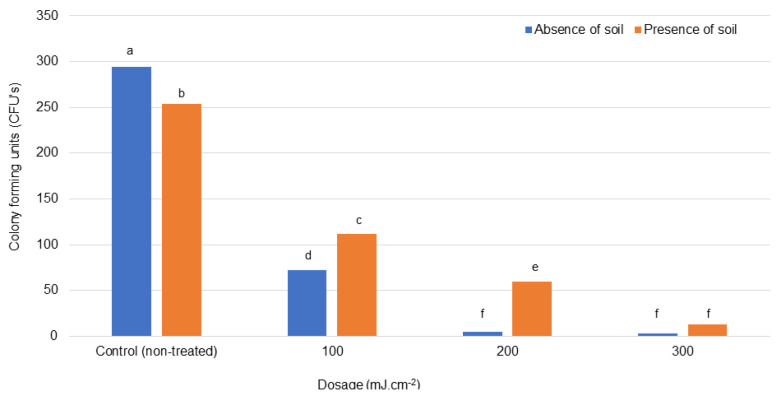
The treatment of *Fusarium oxysporum* f. sp. *cubense*-contaminated water in the presence and absence of soil with ultraviolet radiation. Significant differences (*p* ≤ 0.05) according to Tukey’s test are indicated by different letters.

UV was not able to eradicate Foc in water, even at a dosage of 300 mJ·cm^−2^ (Figure 5). It did, however, significantly reduce the number of CFUs when dosages were increased, both when soil was absent and present. Significantly more Foc CFUs, however, survived in the presence of soil compared to CFUs in clean water (Figure 5).

## 4. Discussion

This study demonstrated that Foc can survive in water for up to 120 days and that agitation plays an important role in the survival of the fungus in water. This agrees with previous studies that indicated that Foc chlamydospores have survived for up to three months in soil submerged in water [13,14,45]. Aeration is possibly the most likely reason why Foc could survive longer in agitated water. Fungi usually require oxygen to survive and under prolonged anaerobic conditions spores will become non-viable [14]. It is thus expected that Foc will survive longer in running water sources like rivers than in farm dams, where water is stagnant [2].

Most Foc spores settled to the bottom of water buckets over time in this study. Deacon [46] reasoned that Foc spores would settle at the bottom of a dam within two days after introduction. A study on *F. oxysporum* f. sp. *cyclaminis* indicated that microconidia settled at the bottom of water in 70 L containers of an ebb and flow system within a day [47]. In our study, however, it took up to 30 days for the Foc spores to sink to the bottom of water in 20-L buckets. Various factors could influence the rate at which spores move in water. Horikawa et al. [48] suggested that *F. oxysporum* spores are easy to wet, and that flocculation depends on the pH and cation species. The negative charge of *F. oxysporum* conidia are decreased by positively charged clay particles in water, which results in mutual flocculation. Other factors not present under laboratory conditions such as solar radiation [49], extreme temperatures and pH [50], and the presence of aquatic biota [51] could further affect the survival of Foc in environmental water. The impact of these factors should be investigated further. Nevertheless, the finding that Foc spores sank to the bottom in water has implications for its management and prompted Deacon [46] to suggest that irrigation water should be extracted from the surface of dams. Based on the time that Foc survived on the surface of water in this study, it is recommended that irrigation water from rivers and farm dams be treated before it is used in plantations.

The eradication of plant pathogens from irrigation water poses a significant challenge. Rainwater is pathogen-free, but rivers, dams and ponds used for sprinkler and flood irrigation may be contaminated if the water had been in contact with Foc-infested soil. Using water from such sources can result in the contamination of disease-free areas [10]. Similarly, drainage and flood waters flowing from Foc-contaminated fields into disease-free plantations can also introduce the pathogen. Irrigation and flood water, therefore, must be carefully managed to prevent the introduction and spread of Foc in disease-free areas. For sprinkler irrigation, this means the treatment of water before irrigation, and for flood irrigation and flooding it means the proper planning of topologies and position of drainage channels.

In the presence of soil, chlorine and the PAA product were ineffective, and the action of UV and ozone was significantly reduced. Organic material usually decreases the contact time between the treatment and the pathogen [52]. When applying oxidising agents, free actives such as chlorine and PAA are usually consumed by organic material in solution [53]. The presence of ions, such as iron and manganese that are present in organic matter in the soil, is known to reduce the efficacy of ozone [54]. Particulate material creates a physical batter between UV and the pathogen [18,22]. Higher dosages and longer exposure times are therefore required for the eradication of pathogens in water when organic material is present [31]. It is recommended that organic material be removed by filtration to improve the efficacy of water treatment plants. This could be implemented before water is diverted into a holding dam used for irrigation. It would, however, require installation, which has cost implications.

An effective water treatment method should not only be effective against the target pathogen, but needs to be practical, affordable and not be harmful to the environment. Ozone and UV would require the installation of expensive treatment plants [31]. For large-scale use, a corona discharge ozone generator can be used to covert oxygen by splitting O_2_ molecules into O- atoms to create ozone [31]. Disinfection by UV radiation requires a low (290–315 nm) or medium pressure (220–280 nm) lamp, which provides intensities that can kill bacteria and fungi [55]. From our study, however, UV would be impractical for field application, as doses required to eliminate Foc need a minimum waiting time of 45 min. PAA products and chlorine require the continuous purchase of chemicals, but is readily available and inexpensive [33,39]. Continuous application of chlorine can produce DBP, which is harmful to the environment. PAA products are more environmentally friendly and would, therefore, be preferred as a water treatment product [56].

## 5. Conclusions

This study indicates that Foc can survive in water for more than 120 days. Aeration and agitation appear to play a big part in its survival, which means that running water allows Foc to survive longer than farm dams, where water is stagnant. Foc spores tend to settle at the bottom of water vessels. To reduce the risk of spread of Foc, surface water could be extracted from rivers, streams and dams and treated before irrigation. As organic material lowers the efficacy of water treatments, it is important to completely disinfect irrigation water to prevent the dispersal of Foc [5]. The PAA product would be recommended to treat Foc-contaminated water as it does not require the installation of expensive treatment plants and is regarded as environmentally friendly.

## Data Availability

Data is contained within the article or supplementary material.

## Supplementary Materials

The following are available online at https://www.mdpi.com/article/10.3390/jof7100796/s1, Figure S1: The experimental setup to test for the survival of *Fusarium oxysporum* f. sp. *cubense* in water.
