# Supplementary figures and images for: The Survival and Treatment of Fusarium oxysporum f. sp. cubense in Water
Source: J Fungi (Basel). 2021 Sep 24;7(10):796. doi: 10.3390/jof7100796 (PMC8539256; doi:10.3390/jof7100796)

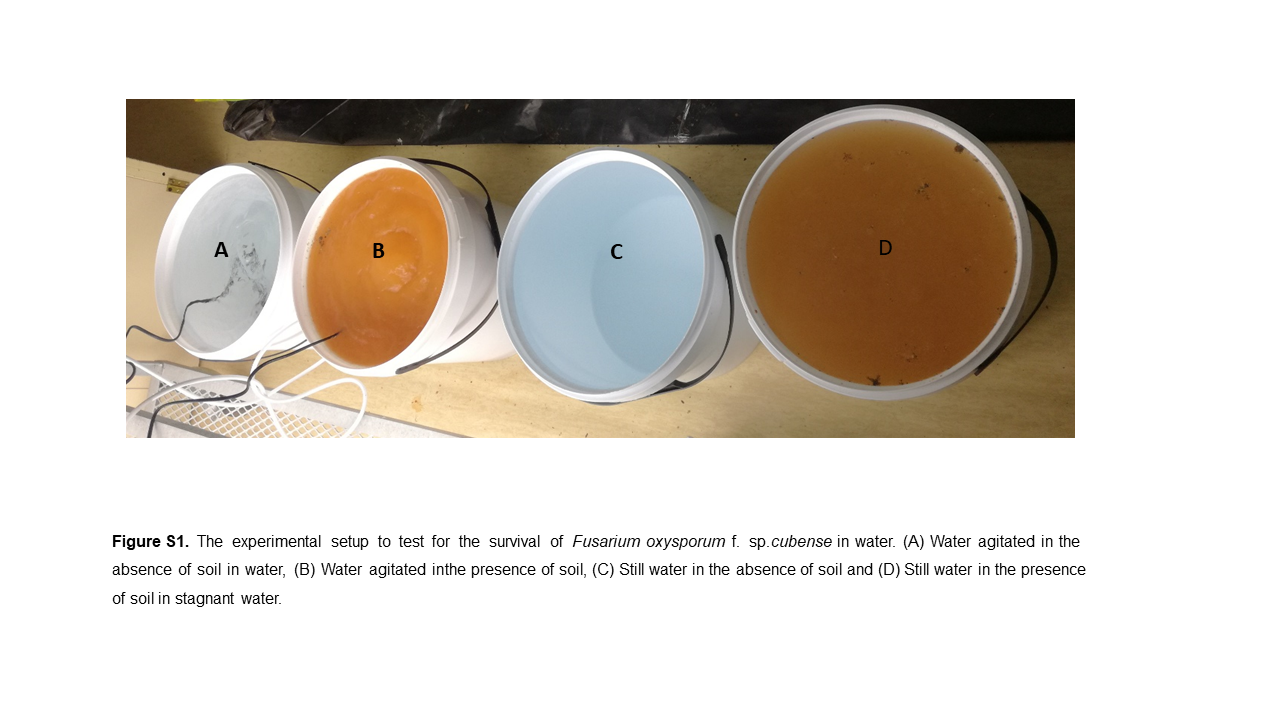

Supplement: Supplementary file 1 [file jof-07-00796-s001.zip › jof-1388134-supplementary.tif]
